# Correlation and Risk Assessment of Inflammation-Based Parameters on Cardiovascular Parameters and Clinical Events in Giant Cell Arteritis: A Retrospective Study

**DOI:** 10.3390/ijms26147016

**Published:** 2025-07-21

**Authors:** Leyla Schweiger, Andreas Meinitzer, Dieter Szolar, Marianne Brodmann, Christian Dejaco, Franz Hafner, Philipp Jud

**Affiliations:** 1 Division of Angiology, Department of Internal Medicine, Medical University of Graz, 8036 Graz, Austria; marianne.brodmann@medunigraz.at (M.B.); ordination@graz-internist.at (F.H.); philipp.jud@medunigraz.at (P.J.); 2 Institute of Medical and Chemical Laboratory Diagnostics, Medical University of Graz, 8036 Graz, Austria; andreas.meinitzer@medunigraz.at; 3 Diagnostikum Graz Süd-West, 8054 Graz, Austria; graz@diagnostikum.at; 4 Division of Rheumatology, Department of Internal Medicine, Medical University of Graz, 8036 Graz, Austria; christian.dejaco@medunigraz.at; 5 Department of Rheumatology, Hospital of Brunico (SABES-ASDAA), 39031 Brunico, Italy

**Keywords:** giant cell arteritis, cardiovascular risk, inflammation, biomarkers

## Abstract

This study investigated associations of inflammation-based biomarkers with endothelial dysfunction and lipids and their predictive value for clinical outcome parameters in patients with giant cell arteritis (GCA). A total of 138 patients with inactive GCA were retrospectively analyzed to investigate potential differences in inflammatory biomarkers regarding clinical GCA subtypes and potential correlations between inflammatory parameters with markers of endothelial dysfunction and lipid parameters. Additionally, the predictive role of inflammatory biomarkers for clinical outcomes, including disease relapse, all-cause mortality, cardiovascular events, and glucocorticoid adverse effects, was analyzed. GCA individuals without concomitant symptoms of polymyalgia rheumatica and those who received initial glucocorticoid pulse therapy exhibited significantly higher levels of white blood cells and neutrophils (all with *p* < 0.05). No other significant differences were observed between inflammatory biomarkers and clinical GCA subtypes. Additionally, significant correlations were identified between selected inflammation-based ratios and specific markers of endothelial dysfunction and lipid parameters (all with *p* < 0.05). Elevated white blood cells and neutrophils were significant and independent predictors of disease relapse in GCA (all with *p* < 0.05) in multiple logistic regression analysis. No significant associations were found between any other inflammatory biomarker and the occurrence of cardiovascular events, mortality, or glucocorticoid-related adverse effects. In patients with inactive GCA, selected inflammatory parameters correlated with endothelial dysfunction and dyslipidemia and may be predictive of disease relapse.

## 1. Introduction

Giant cell arteritis (GCA), formerly also referred to as temporal arteritis or Horton’s disease, was first described by Bayard Taylor Horton in 1932, although evidence has suggested that its existence dates back to the Renaissance or even earlier [1,2]. GCA is a systemic vasculitis primarily affecting large- and medium-sized arteries, predominantly in individuals over 50 years. Emerging evidence suggests that GCA patients exhibit a significantly increased risk of cardiovascular events, including myocardial infarction, stroke, and peripheral artery disease [3]. GCA is characterized by a persistent inflammatory state that is primarily driven by granulomatous inflammation in the walls of medium and large arteries, involving T cells, macrophages, and several cytokines [4]. This inflammation leads to vascular remodeling, including intimal hyperplasia and neoangiogenesis, and thus, the use of atherosclerotic pathways may be increased in GCA patients [5,6,7]. Furthermore, glucocorticoid therapy, which is a therapeutic hallmark in GCA for controlling inflammation, may exacerbate conventional cardiovascular risk factors, like arterial hypertension, diabetes mellitus, or dyslipidemia [3]. Additionally, many other adverse effects such as osteoporosis, gastritis, or venous thromboembolism (VTE) were associated with glucocorticoid therapy, especially in long-term therapy [7,8,9]. Inflammation is a key player in the pathogenesis of GCA as well as in cardiovascular disease. Additionally, endothelial dysfunction may be another mechanism linking chronic inflammation with the increased risk of cardiovascular events in GCA. Endothelial dysfunction can be assessed by many different parameters, including flow-mediated dilation or asymmetric (ADMA) and symmetric dimethylarginine (SDMA), indicating a dysfunction of nitric oxide pathways. Despite its potential significance, the association between inflammatory biomarkers and endothelial dysfunction in GCA has not been fully elucidated. Additionally, dyslipidemia is another important factor contributing to vascular changes in both GCA and cardiovascular diseases [10,11,12].

Inflammatory parameters, such as C-reactive protein (CRP) and erythrocyte sedimentation rate (ESR), are important markers in diagnosing and managing GCA. Both markers are used and part of the recent European Alliance of Associations for Rheumatology (EULAR)/American College of Rheumatology (ACR) criteria, and can rise before clinical relapse, but they are not always elevated during relapses or active phase [13,14,15]. Neither CRP nor ESR is specifically used as a clinical predictor in GCA, and other inflammatory parameters, like inflammation-based ratios, have hardly been analyzed in GCA. The neutrophil-to-lymphocyte ratio (NLR), the platelet-to-lymphocyte ratio (PLR), and the lymphocyte-to-monocyte ratio (LMR) have been linked to an increased severity of cardiovascular disease and worse outcomes in coronary artery disease [16]. Regarding GCA, high NLR and PLR values have been suggested to be supportive markers for diagnosis and may indicate an increased inflammatory state, but their predictive value needs to be elucidated [17]. To the best of our knowledge, data on other inflammation-based ratios in GCA are still missing.

The present study aims to evaluate potential differences in inflammatory biomarkers in clinical GCA subtypes, potential correlations of inflammatory biomarkers on endothelial dysfunction and lipid metabolism, and the predictive value of inflammatory biomarkers for disease relapse, cardiovascular events, all-cause mortality, and glucocorticoid-related adverse effects in patients with GCA.

## 2. Results

### 2.1. Inflammatory Biomarkers at Study Inclusion

In total, 138 patients with diagnosed GCA were included in this study, of whom 39 (28.3%) had a positive temporal artery biopsy. All measured inflammatory biomarkers and their respective ratios at the time of study inclusion are listed in Table 1. Patient characteristics at study inclusion and outcome parameters during the follow-up period are listed in Supplementary Tables S1 and S2.

### 2.2. Differences in Inflammatory Biomarkers Between GCA Subgroups

A comparative analysis of GCA subgroups demonstrated that patients without polymyalgia rheumatic (PMR) symptoms have significantly higher white blood cell (WBC) (8.20 vs. 7.52 × 10^9^/L, *p* = 0.017) and neutrophil counts (5.6 vs. 4.4 × 10^9^/L, *p* = 0.009) compared to those with PMR. GCA patients who received initial glucocorticoid pulse therapy at diagnosis also had significantly higher WBC (8.35 vs. 7.63 × 10^9^/L, *p* = 0.021) and neutrophil counts (5.7 vs. 4.7 × 10^9^/L, *p* = 0.015) than those who did not receive glucocorticoid pulse therapy. No statistically significant differences in inflammatory biomarkers were observed between GCA patients with and without ocular involvement or between patients stratified by high versus non-high European Society of Cardiology (ESC) risk. Further details regarding the comparative analysis are provided in Table 2.

### 2.3. Correlations Between Parameters of Endothelial Dysfunction, Lipids, and Aortic Diameters with Inflammatory Biomarkers

SDMA demonstrated consistently significant correlations with several inflammatory biomarkers. Positive correlations were observed between SDMA and monocyte count (r = 0.147, *p* = 0.040), NLR (r = 0.156, *p* = 0.029), PLR (r = 0.169, *p* = 0.016), CRP (r = 0.259, *p* < 0.001), erythrocyte sedimentation rate (ESR) (r = 0.280, *p* < 0.001), CRP-to-albumin ratio (CAR) (r = 0.274, *p* < 0.001), fibrinogen (r = 0.246, *p* = 0.009), D-dimer (r = 0.267, *p* < 0.001), fibrinogen-to-albumin ratio (FAR) (r = 0.326, *p* < 0.001), and D-dimer-to-fibrinogen ratio (DFR) (r = 0.244, *p* = 0.011). In contrast, LMR demonstrated negative correlations with SDMA (r = −0.239, *p* < 0.001) and also with the infrarenal abdominal aortic diameter (r = −0.162, *p* = 0.029). Infrarenal abdominal aortic diameter correlated positively with monocyte counts (r = 0.171, *p* = 0.021) and DFR (r = 0.348, *p* < 0.001). Additionally, a positive correlation was observed between ADMA and FAR (r = 0.235, *p* = 0.014). No other significant correlations were found between the inflammatory biomarker and the remaining parameters of endothelial dysfunction or aortic diameter. The complete correlation matrix between parameters of endothelial dysfunction, aortic diameter, and inflammatory parameters is presented in Table 3.

Consistently negative correlations were found between CRP, ESR, CAR, and FAR with total cholesterol, intermediate-density lipoprotein (IDL), high-density lipoprotein (HDL), apolipoprotein A1, and HDL particles, except for FAR with IDL. Furthermore, consistently negative correlations were observed between fibrinogen and D-dimer with HDL, apolipoprotein A1, very-low-density lipoprotein (VLDL) size, and HDL particles, while DFR correlated negatively only with apolipoprotein A1 and HDL particles. Additionally, negative correlations were observed between PLR with triglycerides, apolipoprotein A1, VLDL size, and HDL particles. Single negative correlations were found between WBC with HDL size, as well as between monocytes with HDL and apolipoprotein A1, mean platelet volume (MPV) with low-density lipoprotein (LDL) particles, NLR with HDL particles, aspartate aminotransferase (AST)/alanine aminotransferase (ALT) ratio with triglycerides and VLDL size, ESR with VLDL size, and CAR with LDL particles. Positive correlations were observed between WBC with VLDL, triglycerides, apolipoprotein B, VLDL size, and large VLDL particles; between lymphocytes with triglycerides, apolipoprotein A1, VLDL size, and HDL particles; between neutrophils with triglycerides, VLDL size, and large VLDL particles; and between LMR with total cholesterol, HDL, apolipoprotein A1, VLDL size, and HDL particles. Single positive correlations were observed between thrombocytes with LDL, MPV with HDL particles, AST/ALT ratio with HDL and HDL size, and ESR/CRP ratio with apolipoprotein A1 and HDL particles. Exact correlation coefficients with corresponding *p*-values for correlations between lipid and inflammatory parameters are provided in Table 4.

### 2.4. Associations Between Inflammatory Biomarkers and Outcome Parameters

Simple logistic regression analysis identified significant associations between WBC and neutrophil count with newly developed relapse (OR: 26.50 [95% CI: 3.55–198.08], *p* = 0.001; OR: 7.21 [95% CI: 1.73–30.15], *p* = 0.007, respectively). No other inflammatory biomarker demonstrated statistically significant associations with cardiovascular events or glucocorticoid adverse effects, while FAR revealed a trend toward an increased risk of death (OR: 17.86, 95% CI: 0.67–468.78, *p* = 0.086). In multiple regression analysis, WBC and neutrophils remained as significant predictors for newly developed relapse (OR: 23.60 [95% CI: 3.07–181.16], *p* = 0.002; OR: 6.97 [95% CI: 1.64–29.57], *p* = 0.008, respectively). Details of the simple regression analyses are presented in Table 5.

## 3. Discussion

In this retrospective study, we evaluated inflammatory parameters in patients with GCA during a phase of inactive disease to identify their potential role as discriminative biomarkers among GCA subtypes and as predictors of clinical outcome, with possible links to additional pathophysiological cardiovascular pathways. While all patients were in an inactive phase of GCA, NLR levels were elevated compared to the previously reported cut-off value of 2.417 for patients with Takayasu arteritis [18]. In contrast, PLR and CAR values were within normal ranges described for Takayasu arteritis [18,19]. Notably, there is evidence that NLR may be more sensitive than PLR in reflecting low-grade inflammation or vascular immune activation [17,20]. Although median values of classical inflammatory parameters, like WBC, neutrophils, CRP, and ESR, were within normal ranges in our GCA cohort, the elevated level of NLR suggested subclinical inflammation despite the clinical remission of GCA. This phenomenon has already been described in other autoimmune conditions, like systemic lupus erythematosus, where standard inflammatory markers normalize despite ongoing immune activation reflected by NLR or PLR [21]. In GCA, Galli et al. [22] demonstrated that a significant proportion of GCA patients were revealed to have persistent vascular inflammation on positron emission tomography, even in the absence of clinical symptoms and with normal CRP levels. Although no specific thresholds for other inflammation-based ratios have been established in large-vessel vasculitis yet, the median values of other inflammation-based ratios, like the ESR/CRP ratio with 2.35 and the AST/ALT ratio with 1.20, were also higher in our GCA cohort, suggesting residual or subclinical inflammatory activity, despite clinical remission. An ESR/CRP ratio of 2.0–2.4 may favor autoimmune flare over infection, and a higher ESR/CRP ratio has been correlated with immune-mediated inflammation in systemic lupus erythematosus [23]. Similarly, values of AST/ALT ratios above 1.0 have been associated with systemic inflammation and worse outcomes in rheumatoid arthritis [24]. Furthermore, although fibrinogen levels were within normal limits, both D-dimer and the FAR were elevated in our patient cohort. While elevated D-dimer is non-specific and frequently observed in older individuals and in patients with aortic aneurysms, both are represented in our cohort; an increased FAR may reflect ongoing thromboinflammatory processes [25,26].

Inflammation-based ratios did not effectively differentiate between clinical subtypes of GCA in our cohort, whereas some conventional inflammatory parameters demonstrated significant differences with higher WBC and neutrophil counts in patients without concomitant PMR and in those who had received initial high-dose glucocorticoid pulse therapy. Differences in the PMR subtype may be explained by the fact that PMR-negative GCA is characterized by myeloid dominance with enhanced bone marrow activity, while PMR-positive GCA may involve lymphoid–myeloid interactions that activate neutrophil-suppressing mechanisms by interleukins [4,27]. The tendency toward higher NLR in PMR-negative GCA patients may reflect subclinical, neutrophil-driven inflammation, although statistical significance was not achieved in our cohort [28]. This differential leukocyte profile raises the possibility of underlying genetic factors influencing hematopoietic lineage commitment. However, to date, there is limited direct evidence in the literature identifying specific genetic variants that modulate bone marrow responses to glucocorticoid therapy in GCA or similar conditions. Polymorphisms in genes regulating hematopoietic stem cell differentiation and cytokine signaling pathways have been hypothesized to predispose individuals to a myeloid- or lymphoid-dominant immune response. Similarly, genomic pathways could potentially explain results on initial high-dose glucocorticoid pulse therapy. In our cohort, GCA patients who had received high-dose glucocorticoid pulse therapy exhibited persistently elevated WBC and neutrophil counts, even several years after treatment initiation. This observation may reflect the enduring effects of steroid-induced leukocytosis. While acute leukocytosis following glucocorticoid administration is well documented and mediated by neutrophil demargination, reduced apoptosis, and increased bone marrow egress, the long-term persistence of these hematological alterations suggests more profound changes in hematopoietic regulation [29]. Emerging research indicates that high-dose or prolonged glucocorticoid therapy can induce epigenetic reprogramming and transcriptional shifts in hematopoietic stem and progenitor cells, resulting in a sustained bias toward myelopoiesis, even in the absence of ongoing systemic inflammation or active glucocorticoid exposure. Studies have shown that glucocorticoids can impair the development of innate lymphoid cells from hematopoietic stem cells, favoring myeloid lineage differentiation [30]. Moreover, individual genetic predispositions may modulate the extent and duration of these effects. Nevertheless, the role of individual genetic predispositions remains largely theoretical, as robust clinical evidence is currently lacking. Variants in genes encoding the glucocorticoid receptor, cytokines, or growth factors have been suggested to play a role in differential steroid sensitivity and hematopoietic lineage commitment, but further studies are needed to substantiate these associations in the context of GCA [31]. The unbalanced comparison between GCA patients with and without ocular involvement, as well as those patients with and without high ESC risk, may be explained by the lack of significant results for inflammatory parameters in these GCA subtypes.

In correlation analysis, significant correlations between several inflammatory parameters and SDMA were observed, suggesting that subclinical inflammatory processes may affect arginine metabolism by SDMA. Notably, recent advances in GCA pathogenesis have shown that vessel wall-resident cells, particularly vascular smooth muscle cells (VSMCs), actively participate in vascular inflammation and remodeling by responding to inflammatory stimuli and interacting with innate immune cells [32]. This interaction promotes the phenotypic switching of VSMC, leading to an increased production of pro-inflammatory mediators and matrix-remodeling enzymes, which contribute to vascular remodeling in GCA. Potential linkage between VSMC and nitric oxide-dependent endothelial dysfunction has been described, and therefore, SDMA-induced endothelial dysfunction and altered arginine metabolism could further influence VSMC activation [33]. However, to the best of our knowledge, such a linkage between SDMA and VSMC has not been described so far in GCA. High NLR and PLR have been associated with worse vascular outcomes and increased arterial stiffness, and their correlation with fibrinogen, D-dimer, FAR, and DFR implies thromboinflammatory processes that are potentially mediated by endothelial adhesion molecules that enhance leukocyte transmigration [34,35,36,37]. The negative correlation between LMR and SDMA highlights again a potential myeloid dominance as SDMA may exacerbate this imbalance by depleting arginine and impairing T-cell function, while monocytes propagate inflammation via CX3CR1-CX3CL1/CCR2-CCL2 pathways [36,37]. However, other assessed parameters of endothelial dysfunction revealed no significant correlations with inflammatory parameters. The observed positive correlations between selected inflammatory parameters with SDMA support the concept that subclinical inflammation and endothelial dysfunction may be interlinked, but the overall interaction seems to be marginal. On the other hand, the number of significant lipid–inflammatory correlations in GCA patients was higher compared to the correlations between inflammation and endothelial dysfunction. However, overall, they were also more inconsistent. Some correlations between selected parameters may be explained by inflammation-driven dyslipidemia, which is characterized by lower HDL and higher levels of LDL and triglycerides [38]. In our cohort, HDL, HDL particles, and apolipoprotein A1 were consistently and negatively correlated with CRP, ESR, CAR, or FAR, while WBC, neutrophils, and lymphocytes were positively correlated with triglycerides. However, there were also missing and paradoxical correlations between lipids and inflammatory parameters observed, if it is, in fact, true that inflammation-driven dyslipidemia is the main cause for the changes. LDL and apolipoprotein B correlated only with thrombocytes and WBC, LDL size correlated with no inflammatory parameter, and NLR revealed a single correlation with HDL particles. Additionally, although CRP and ESR showed consistent negative correlations with HDL, HDL particles, and apolipoprotein A1, the ESR/CRP ratio revealed positive correlations with HDL particles and apolipoprotein A1. One possible explanation may be the absence of multiple tests, and especially, single significant correlations may be incidental findings. Nevertheless, the potential interactions between lipids and inflammation in inactive GCA remain inconclusive, and larger studies are needed.

Several studies reported an association between inflammatory parameters and cardiovascular mortality, and their predictive value can vary depending on the studied population and the specific assessed outcome [39,40,41]. CRP and other acute-phase reactants have been robustly linked to increased cardiovascular risk, but these associations may be attenuated after adjustment for established risk factors or in studies with smaller sample sizes [40,41]. In contrast to these broader findings, the present study did not observe significant associations between inflammatory parameters and cardiovascular events, mortality, or glucocorticoid adverse effects. Especially, inflammation-based ratios failed as potential outcome predictors. This may be attributed to limited statistical power, a relatively small sample size, and the fact that confounding variables are not accounted for in simple regression models. The strong association of WBC and neutrophil counts with relapse, even during inactive disease phases, suggests that these markers may be more reflective of immunological activity relevant to disease recurrence rather than chronic vascular risk.

This study has several limitations. First, due to its retrospective design, the potential for selection bias and unmeasured confounders cannot be excluded. Second, all biomarker measurements were performed at a single time point during clinical remission, which may not fully reflect dynamic changes over time, especially during the active phase. Third, the relatively small sample size and lack of adjustment for established cardiovascular risk factors in multiple regression analysis limit the interpretability of the predictive analyses. Fourth, detailed documentation of ongoing glucocorticoid therapy after treatment initiation within GCA patients was not available, as this information was not systematically recorded in the recent study. Finally, the exploratory approach and absence of correction for multiple testing increase the risk of type I error. Nevertheless, our study is among the few to comprehensively assess inflammatory biomarkers, endothelial dysfunction, and lipid metabolism in a well-characterized GCA cohort.

## 4. Materials and Methods

### 4.1. Study Design and Patient Cohort

This study is a sub-study of a prior published study by Jud et al. [42]. Patients with GCA, who were diagnosed between 1993 and 2010, were identified by electronic medical records and invited to participate in the study in 2012. Experienced angiologists or rheumatologists established GCA diagnosis based on clinical presentation, laboratory findings, imaging studies, and/or biopsy results. All patients retrospectively fulfilled the modified ACR criteria as proposed by Dejaco et al. [43]. During the baseline assessment period (January–December 2012), comprehensive evaluations, including blood sampling for lipid metabolism, endothelial dysfunction, and inflammatory biomarkers, were performed. Additionally, ultrasonography and pulse-wave analysis measuring intima-media thickness (IMT) and arterial stiffness, and computed tomography (CT) evaluated aortic pathology. Patients were subsequently followed up with through routine clinical visits. In 2020, a detailed chart review was conducted to collect pre-study clinical, radiological, and laboratory data and to report cardiovascular events, mortality, GCA relapses, and potential glucocorticoid adverse effects occurring after study inclusion. To ensure an adequate observation period for outcome parameters, only patients with a GCA diagnosis at least two years prior to recruitment were included. All assessments were conducted during inactive disease phases, with no relapses reported within six months prior to participation. Exclusion criteria included active malignancies, ongoing infections, or other forms of vasculitis. GCA patients were subdivided into clinical subgroups to investigate potential differences in inflammatory parameters among these subgroups. GCA subgroups included patients with or without PMR, those with or without initial ocular involvement, those with high or non-high ESC risk, and those with or without initial glucocorticoid pulse therapy. ESC risk SCORE2/SCORE2-OP was calculated post hoc based on clinical and laboratory data at study inclusion according to recent guidelines [44].

### 4.2. Vascular Assessment

Detailed methods measuring IMT, arterial stiffness, and aortic diameters have been previously described [42]. In brief, the IMT of the carotid, subclavian, and femoral arteries was measured by ultrasonography, considering IMT ≥ 0.9 mm in any common carotid artery to be pathological. Arterial stiffness parameters, including carotid–femoral pulse-wave velocity (PWV) and the augmentation index (Aix), were assessed using the Vascular Explorer device, while PWV values > 10 m/s were classified as abnormal [45]. Aortic diameters were assessed using contrast-enhanced multidetector CT of the thoracic and abdominal regions, measuring manually from outer wall to outer wall in the anteroposterior plane. Aortic dilatation or aneurysm was defined as a diameter exceeding the 90th percentile for the respective aortic segment, which was adjusted for age, sex, and body surface area, based on reference values [46].

### 4.3. Biochemical Analyses

Fasting blood samples were collected prior to CT imaging, evaluating parameters of inflammation, endothelial dysfunction, and lipids. The following inflammatory biomarkers were analyzed: WBC, neutrophils, lymphocytes, monocytes, thrombocytes, MPV, ESR, fibrinogen, CRP, D-dimer, ALT, AST, and albumin. Derived inflammation-based ratios included NLR, LMR, PLR, the AST/ALT ratio, the ESR/CRP ratio, CAR, FAR, and DFR. These inflammatory parameters were measured by routine laboratory work-up and were calculated post hoc. Additionally, ADMA and SDMA were measured as biochemical parameters for endothelial dysfunction, and total cholesterol, VLDL, IDL, LDL, HDL, triglyceride, lipoprotein (a), apolipoprotein A1, and apolipoprotein B were measured as lipid parameters. Detailed methodologies for the measurement of endothelial dysfunction and lipid metabolism parameters have been previously described [47].

### 4.4. Chart Review of Outcome Parameters

A comprehensive chart review was conducted in 2020 using the MEDOCS electronic health system in Styria, Austria, recording outcome parameters. Outcome parameters were defined as newly developed cardiovascular events, death, glucocorticoid adverse effects, and relapse of GCA during the observational period. Cardiovascular events were categorized as coronary artery disease (CAD), carotid and vertebral artery disease (CVAD), upper extremity arterial disease (UEAD), renal artery disease (RAD), mesenteric artery disease (MAD), and lower extremity artery disease (LEAD) in accordance with the ESC guidelines from 2017 [44]. Potential adverse effects of glucocorticoid therapy were defined as arterial hypertension, diabetes mellitus, obesity, hyperlipidemia, including hypercholesterolemia and hypertriglyceridemia, chronic kidney disease, osteoporosis, bone fracture, cataract, glaucoma, hepatic steatosis and cirrhosis, venous thromboembolism, depression, dementia, gastritis, peptic ulcer, esophagitis, and pancreatitis. Relapse was defined as major or minor according to the EULAR recommendations for the management of large-vessel vasculitis [13]. Additionally, patients’ demographics, cardiovascular diseases, and potential prevalent glucocorticoid adverse effects prior to study inclusion were also recorded. Further details about the record of outcome parameters have been previously described [41,47].

### 4.5. Statistics

Statistical analyses were conducted using SPSS v28.0, with significance set at *p* < 0.05. Categorical variables were presented as frequencies and percentages, while normally distributed continuous variables were expressed as means ± standard deviations and non-normally distributed variables as medians with interquartile ranges. Normality of distribution was assessed using the Kolmogorov–Smirnov test and visual inspection. Group comparisons were performed using χ^2^ tests for categorical variables, independent *t*-tests for normally distributed data, and Mann–Whitney U tests for non-normally distributed data. Pearson’s and Spearman’s correlation coefficients were used for normally and non-normally distributed variables, respectively. A simple logistic regression analysis was performed to assess associations between inflammatory biomarkers and outcome parameters. Multiple regression analysis was adjusted for confounding variables, including age and sex. Given the exploratory nature of the study, no correction for multiple testing was applied.

## 5. Conclusions

In conclusion, our findings demonstrate that patients with GCA exhibit evidence of subclinical inflammation even during clinical remission, which may interact with endothelial dysfunction and dyslipidemia. The observed associations between WBC and neutrophil counts and disease relapse suggest a prominent role for myeloid-driven inflammation in disease recurrence, underscoring the need for integrated biomarker strategies to optimize individualized management in GCA.

## Figures and Tables

**Table 1:** Inflammatory parameters and ratios at study inclusion.

	GCA (*n* = 138)
WBC (10^9^/L), median (25th–75th percentile)	7.91 (6.21–9.43)
Monocytes	0.5 (0.4–0.7)
Lymphocytes	1.5 (1.2–2.0)
Neutrophils	5.0 (3.9–6.7)
Thrombocytes (10^9^/L), median (25th–75th percentile)	246 (208–293)
MPV (fL), median (25th–75th percentile)	10.6 (9.9–11.3)
NLR, median (25th–75th percentile)	3.3 (2.3–5.1)
LMR, median (25th–75th percentile)	3.0 (2.0–3.8)
PLR, median (25th–75th percentile)	158.6 (124.3–211.6)
AST/ALT ratio, median (25th–75th percentile)	1.20 (1.00–1.48)
CRP (mg/L), median (25th–75th percentile)	3.5 (1.8–7.5)
ESR (mm/h), median (25th–75th percentile)	10 (6–17)
ESR/CRP ratio, median (25th–75th percentile)	2.35 (1.29–5.00)
CAR, median (25th–75th percentile)	0.83 (0.43–1.74)
Fibrinogen (mg/dL), median (25th–75th percentile)	320 (289–354)
D-dimer (mg/L), median (25th–75th percentile)	0.70 (0.45–1.08)
FAR, median (25th–75th percentile)	74.22 (65.65–84.61)
DFR, median (25th–75th percentile)	0.000187 (0.000115–0.000354)

Abbreviations: AST/ALT: aspartate aminotransferase/alanine aminotransferase; CAR: C-reactive protein/albumin ratio; CRP: C-reactive protein; DFR: D-dimer/fibrinogen ratio; ESR: erythrocyte sedimentation rate; FAR: fibrinogen/albumin ratio; GCA: giant-cell arteritis; LMR: lymphocyte/monocyte ratio; MPV: mean platelet volume; NLR: neutrophil/lymphocyte ratio; PLR: platelet/lymphocyte ratio; WBCs: white blood cells.

**Table 2:** Differences in inflammatory parameters and ratios between GCA subgroups.

	GCA With PMR (*n* = 61)	GCA Without PMR (*n* = 77)	*p*-Value
WBC (10^9^/L), median (25th–75th percentile)	7.52 (5.81–9.13)	8.20 (7.18–9.63)	**0.017**
Monocytes	0.6 (0.4–0.7)	0.5 (0.4–0.7)	0.855
Lymphocytes	1.5 (1.2–2.0)	1.5 (1.1–2.0)	0.920
Neutrophils	4.4 (3.5–6.6)	5.6 (4.4–6.8)	**0.009**
Thrombocytes (10^9^/L), median (25th–75th percentile)	246 (202–291)	244 (209–294)	0.692
MPV (fL), median (25th–75th percentile)	10.6 (10.0–11.4)	10.6 (9.9–11.3)	0.886
NLR, median (25th–75th percentile)	2.9 (2.1–4.4)	3.5 (2.4–5.7)	0.090
LMR, median (25th–75th percentile)	3.1 (2.2–4.0)	3.0 (2.0–3.8)	0.701
PLR, median (25th–75th percentile)	146.3 (121.7–203.9)	164.6 (126.7–218.3)	0.344
AST/ALT ratio, median (25th–75th percentile)	1.24 (1.04–1.40)	1.20 (0.95–1.50)	0.588
CRP (mg/L), median (25th–75th percentile)	3.0 (1.6–7.4)	3.9 (2.0–7.6)	0.397
ESR (mm/h), median (25th–75th percentile)	9 (6–16)	10 (7–18)	0.446
ESR/CRP ratio, median (25th–75th percentile)	2.55 (1.18–5.41)	2.28 (1.45–4.44)	0.948
CAR, median (25th–75th percentile)	0.74 (0.35–1.78)	0.94 (0.48–1.73)	0.432
Fibrinogen (mg/dL), median (25th–75th percentile)	311 (287–340)	336 (290–358)	0.229
D-dimer (mg/L), median (25th–75th percentile)	0.58 (0.45–1.01)	0.74 (0.43–1.12)	0.406
FAR, median (25th–75th percentile)	73.22 (65.33–84.74)	74.22 (65.40–84.93)	0.643
DFR, median (25th–75th percentile)	0.000192 (0.000115–0.000338)	0.000147 (0.000108–0.000378)	0.534
	**GCA With Ocular Involvement (*n* = 12)**	**GCA Without Ocular Involvement (*n* = 126)**	***p*-Value**
WBC (10^9^/L), median (25th–75th percentile)	7.66 (5.83–8.39)	7.91 (6.29–9.51)	0.357
Monocytes	0.5 (0.4–0.6)	0.5 (0.4–0.7)	0.275
Lymphocytes	1.3 (1.1–1.8)	1.5 (1.2–2.0)	0.212
Neutrophils	5.1 (3.4–6.3)	5.0 (3.9–6.7)	0.433
Thrombocytes (10^9^/L), median (25th–75th percentile)	218 (193–250)	248 (208–295)	0.078
MPV (fL), median (25th–75th percentile)	10.8 (10.5–11.3)	10.6 (9.9–11.4)	0.343
NLR, median (25th–75th percentile)	3.4 (2.0–5.7)	3.2 (2.3–4.9)	0.784
LMR, median (25th–75th percentile)	3.0 (2.0–3.4)	3.0 (2.0–3.8)	0.592
PLR, median (25th–75th percentile)	161.0 (117.1–281.1)	158.5 (125.1–211.6)	0.925
AST/ALT ratio, median (25th–75th percentile)	1.21 (0.95–1.71)	1.20 (1.00–1.45)	0.761
CRP (mg/L), median (25th–75th percentile)	2.8 (1.0–6.2)	3.5 (2.0–7.5)	0.257
ESR (mm/h), median (25th–75th percentile)	8 (7–21)	10 (6–17)	0.709
ESR/CRP ratio, median (25th–75th percentile)	3.21 (1.67–9.00)	2.35 (1.26–5.00)	0.507
CAR, median (25th–75th percentile)	0.64 (0.23–1.55)	0.84 (0.47–1.78)	0.292
Fibrinogen (mg/dL), median (25th–75th percentile)	334 (298–354)	317 (288–363)	0.845
D-dimer (mg/L), median (25th–75th percentile)	1.00 (0.55–1.39)	0.69 (0.44–1.05)	0.158
FAR, median (25th–75th percentile)	74.22 (63.40–80.68)	74.28 (65.98–86.86)	0.572
DFR, median (25th–75th percentile)	0.000328 (0.000133–0.000510)	0.000177 (0.000109–0.000347)	0.241
	GCA With High ESC Risk (*n* = 102) *	GCA Without High ESC Risk (*n* = 34) *	***p*-Value**
WBC (10^9^/L), median (25th–75th percentile)	7.91 (6.09–9.43)	7.86 (6.15–9.20)	0.710
Monocytes	0.5 (0.4–0.7)	0.5 (0.4–0.7)	0.787
Lymphocytes	1.5 (1.2–2.0)	1.6 (1.3–2.0)	0.840
Neutrophils	5.0 (3.8–6.7)	5.0 (4.0–6.8)	0.957
Thrombocytes (10^9^/L), median (25th–75th percentile)	243 (204–289)	260 (229–297)	0.229
MPV (fL), median (25th–75th percentile)	10.7 (10.0–11.4)	10.5 (9.8–11.2)	0.161
NLR, median (25th–75th percentile)	3.1 (2.4–4.9)	3.2 (2.2–5.5)	0.944
LMR, median (25th–75th percentile)	3.0 (2.0–3.7)	3.3 (2.0–4.0)	0.624
PLR, median (25th–75th percentile)	153.6 (124.0–211.6)	171.8 (125.1–209.9)	0.397
AST/ALT ratio, median (25th–75th percentile)	1.19 (0.99–1.46)	1.25 (1.16–1.53)	0.133
CRP (mg/L), median (25th–75th percentile)	3.3 (1.7–6.3)	3.9 (2.1–9.0)	0.235
ESR (mm/h), median (25th–75th percentile)	10 (7–18)	8 (5–17)	0.544
ESR/CRP ratio, median (25th–75th percentile)	2.56 (1.45–5.83)	1.94 (1.17–3.37)	0.077
CAR, median (25th–75th percentile)	0.80 (0.39–1.61)	0.90 (0.49–2.00)	0.381
Fibrinogen (mg/dL), median (25th–75th percentile)	314 (286–355)	330 (298–352)	0.598
D-dimer (mg/L), median (25th–75th percentile)	0.71 (0.46–1.05)	0.61 (0.39–1.29)	0.883
FAR, median (25th–75th percentile)	74.21 (66.98–84.86)	75.90 (63.73–84.96)	0.862
DFR, median (25th–75th percentile)	0.000195 (0.000124–0.000339)	0.000143 (0.000086–0.000374)	0.150
	**GCA With Initial Glucocorticoid Pulse Therapy (*n* = 31) ^†^**	**GCA Without Initial Glucocorticoid Pulse Therapy (*n* = 60) ^†^**	***p*-Value**
WBC (10^9^/L), median (25th–75th percentile)	8.35 (7.52–9.99)	7.63 (5.93–8.99)	**0.021**
Monocytes	0.5 (0.4–0.7)	0.5 (0.4–0.7)	0.474
Lymphocytes	1.4 (1.1–1.9)	1.7 (1.2–2.1)	0.381
Neutrophils	5.7 (4.8–6.9)	4.7 (3.6–6.4)	**0.015**
Thrombocytes (10^9^/L), median (25th–75th percentile)	233 (197–265)	247 (215–296)	0.118
MPV (fL), median (25th–75th percentile)	10.7 (10.1–11.3)	10.8 (10.0–11.4)	0.887
NLR, median (25th–75th percentile)	4.1 (2.6–6.0)	2.9 (2.2–4.5)	0.061
LMR, median (25th–75th percentile)	2.8 (2.0–3.5)	3.2 (2.3–3.9)	0.230
PLR, median (25th–75th percentile)	160.0 (125.3–221.2)	153.6 (117.1–214.4)	0.847
AST/ALT ratio, median (25th–75th percentile)	1.13 (0.81–1.69)	1.23 (1.00–1.50)	0.419
CRP (mg/L), median (25th–75th percentile)	3.8 (1.6–6.3)	3.1 (1.5–6.3)	0.840
ESR (mm/h), median (25th–75th percentile)	10 (7–18)	10 (5–18)	0.398
ESR/CRP ratio, median (25th–75th percentile)	2.26 (1.55–7.15)	2.70 (1.29–5.40)	0.919
CAR, median (25th–75th percentile)	0.96 (0.39–1.61)	0.71 (0.34–1.58)	0.702
Fibrinogen (mg/dL), median (25th–75th percentile)	336 (291–354)	316 (288–350)	0.662
D-dimer (mg/L), median (25th–75th percentile)	0.93 (0.51–1.31)	0.59 (0.38–1.03)	0.066
FAR, median (25th–75th percentile)	73.74 (70.98–80.35)	74.04 (63.37–85.63)	0.970
DFR, median (25th–75th percentile)	0.000171 (0.000125–0.000333)	0.000203 (0.000112–0.000392)	0.881

Abbreviations: AST/ALT: aspartate aminotransferase/alanine aminotransferase; CAR: C-reactive protein/albumin ratio; CRP: C-reactive protein; DFR: D-dimer/fibrinogen ratio; ESC: European Society of Cardiology; ESR: erythrocyte sedimentation rate; FAR: fibrinogen/albumin ratio; GCA: giant-cell arteritis; LMR: lymphocyte/monocyte ratio; MPV: mean platelet volume; NLR: neutrophil/lymphocyte ratio; PLR: platelet/lymphocyte ratio; PMR: polymyalgia rheumatica; WBCs: white blood cells. *: data from two patients were not included due to missing subparameters for the calculation of ESC SCORE2/-OP; ^†^: data from 47 patients were not included due to missing information in retrospective data collection.

**Table 3:** Correlations between parameters of endothelial dysfunction, lipids, and aortic diameters with inflammatory parameters and ratios.

		Carotid IMT	Femoral IMT	Subclavian IMT	Carotid–Femoral PWV	Aix	ADMA	SDMA	Diameter of Ascending Aorta	Diameter of Thoracic Descending Aorta	Diameter of Infrarenal Abdominal Aorta
**WBC**	r	0.107	−0.041	−0.016	−0.030	−0.003	−0.076	0.123	0.025	−0.065	0.075
	*p*	0.105	0.541	0.812	0.680	0.969	0.282	0.082	0.714	0.335	0.311
**Monocytes**	r	0.059	−0.053	0.002	0.061	0.039	0.076	0.147	0.029	0.054	0.171
	*p*	0.380	0.438	0.980	0.404	0.592	0.288	0.040	0.674	0.428	0.021
**Lymphocytes**	r	−0.011	−0.031	0.047	0.054	0.052	−0.051	−0.100	0.107	0.039	0.003
	*p*	0.871	0.646	0.491	0.458	0.474	0.472	0.159	0.111	0.563	0.969
**Neutrophils**	r	0.058	−0.051	−0.065	−0.070	−0.045	−0.040	0.112	0.033	−0.070	0.070
	*p*	0.389	0.454	0.343	0.338	0.535	0.581	0.118	0.633	0.310	0.346
**Thrombocytes**	r	0.008	−0.123	−0.058	0.142	0.115	0.066	0.110	0.014	−0.021	0.091
	*p*	0.906	0.084	0.395	0.071	0.111	0.350	0.121	0.841	0.753	0.219
**MPV**	r	−0.079	−0.018	0.058	−0.069	−0.067	−0.089	−0.081	0.107	0.065	0.006
	*p*	0.234	0.791	0.394	0.344	0.355	0.208	0.255	0.113	0.338	0.934
**NLR**	r	0.024	−0.042	−0.094	−0.080	−0.075	0.052	0.156	−0.041	−0.081	0.063
	*p*	0.717	0.534	0.173	0.275	0.304	0.471	0.029	0.545	0.237	0.396
**LMR**	r	−0.025	0.016	0.055	−0.020	0.037	−0.139	−0.239	0.026	0.029	−0.162
	*p*	0.709	0.815	0.421	0.781	0.609	0.052	<0.001	0.709	0.670	0.029
**PLR**	r	0.007	−0.056	−0.039	0.028	0.037	0.095	0.169	−0.092	−0.050	0.079
	*p*	0.912	0.405	0.566	0.700	0.610	0.179	0.016	0.171	0.458	0.283
**AST/ALT ratio**	r	0.073	0.023	−0.031	−0.004	0.063	0.011	0.091	−0.093	−0.017	0.079
	*p*	0.274	0.732	0.649	0.951	0.380	0.877	0.200	0.169	0.800	0.284
**CRP**	r	0.090	−0.008	0.034	0.039	−0.070	0.128	0.259	0.015	−0.012	0.110
	*p*	0.179	0.905	0.624	0.597	0.337	0.074	<0.001	0.832	0.859	0.141
**ESR**	r	0.067	−0.030	−0.006	−0.040	−0.048	0.048	0.280	0.001	−0.095	0.056
	*p*	0.345	0.671	0.932	0.602	0.536	0.514	<0.001	0.985	0.187	0.472
**ESR/CRP ratio**	r	−0.113	−0.053	−0.111	−0.047	0.073	−0.090	−0.096	−0.009	−0.097	−0.082
	*p*	0.108	0.451	0.123	0.545	0.345	0.223	0.194	0.901	0.178	0.291
**CAR**	r	0.108	0.030	0.056	0.043	−0.081	0.139	0.274	0.013	−0.011	0.100
	*p*	0.108	0.656	0.419	0.562	0.271	0.055	<0.001	0.849	0.875	0.183
**Fibrinogen**	r	0.045	−0.127	−0.110	0.088	−0.010	0.141	0.246	0.026	−0.115	0.064
	*p*	0.599	0.139	0.209	0.354	0.915	0.138	0.009	0.762	0.189	0.513
**D-dimer**	r	0.043	−0.025	−0.025	0.122	−0.007	0.098	0.267	−0.172	−0.145	0.142
	*p*	0.536	0.723	0.722	0.111	0.926	0.188	<0.001	0.051	0.064	0.066
**FAR**	r	0.112	0.011	−0.040	0.052	−0.105	0.235	0.326	−0.002	−0.095	0.083
	*p*	0.192	0.903	0.650	0.586	0.273	0.014	<0.001	0.978	0.279	0.403
**DFR**	r	0.033	−0.008	0.015	0.113	0.011	0.100	0.244	−0.173	0.005	0.348
	*p*	0.704	0.929	0.871	0.245	0.907	0.305	0.011	0.051	0.955	<0.001

Abbreviations: ADMA: asymmetric dimethylarginine; Aix: augmentation index; AST/ALT: aspartate aminotransferase/alanine aminotransferase; CAR: C-reactive protein/albumin ratio; CRP: C-reactive protein; DFR: D-dimer/fibrinogen ratio; ESR: erythrocyte sedimentation rate; FAR: fibrinogen/albumin ratio; IMT: intima-media thickness; LMR: lymphocyte/monocyte ratio; MPV: mean platelet volume; NLR: neutrophil/lymphocyte ratio; PLR: platelet/lymphocyte ratio; PWV: pulse-wave velocity; SDMA: symmetric dimethylarginine; WBCs: white blood cells.

**Table 4:** Correlations between lipid parameters, inflammatory parameters, and ratios.

		Total Cholesterol	VLDL	LDL	IDL	HDL	Triglycerides	Lipoprotein (a)	Apolipoprotein A1	Apolipoprotein B	VLDL Size	LDL Size	HDL Size	Large VLDL Particles	LDL Particles	HDL Particles
**WBC**	r	0.110	0.190	0.070	0.044	0.002	0.281	−0.007	0.032	0.148	0.207	0.004	−0.131	0.181	0.103	0.027
	*p*	0.089	0.013	0.286	0.527	0.971	<0.001	0.917	0.626	0.026	0.001	0.950	0.044	0.008	0.165	0.684
**Monocytes**	r	−0.080	0.022	−0.024	−0.105	−0.143	0.033	−0.048	−0.153	0.026	0.033	0.001	−0.063	−0.025	−0.024	−0.079
	*p*	0.225	0.780	0.716	0.131	0.030	0.617	0.475	0.022	0.697	0.617	0.986	0.344	0.727	0.746	0.233
**Lymphocytes**	r	0.092	0.076	0.038	0.024	0.045	0.145	−0.092	0.161	0.081	0.147	0.026	−0.096	0.093	−0.007	0.284
	*p*	0.158	0.327	0.561	0.731	0.495	0.026	0.168	0.015	0.224	0.024	0.688	0.142	0.177	0.929	<0.001
**Neutrophils**	r	0.050	0.108	0.015	0.041	0.024	0.197	0.057	0.022	0.086	0.138	−0.005	−0.071	0.138	0.076	−0.040
	*p*	0.454	0.168	0.819	0.555	0.716	0.003	0.402	0.743	0.199	0.037	0.941	0.284	0.048	0.313	0.544
**Thrombocytes**	r	0.062	−0.007	0.129	0.032	0.018	−0.001	0.085	0.014	0.104	−0.069	−0.009	−0.049	−0.044	0.069	−0.016
	*p*	0.338	0.925	0.047	0.646	0.787	0.991	0.205	0.832	0.118	0.295	0.890	0.455	0.527	0.352	0.811
**MPV**	r	0.085	0.035	0.006	−0.041	0.054	0.093	−0.053	0.110	0.069	0.105	−0.022	−0.067	0.080	−0.165	0.143
	*p*	0.196	0.655	0.930	0.549	0.411	0.157	0.428	0.101	0.299	0.108	0.735	0.310	0.249	0.026	0.029
**NLR**	r	−0.036	−0.022	−0.009	−0.017	−0.041	0.026	0.084	−0.124	0.008	−0.023	−0.010	0.029	0.008	0.007	−0.261
	*p*	0.584	0.782	0.898	0.810	0.538	0.690	0.211	0.064	0.903	0.731	0.880	0.663	0.904	0.930	<0.001
**LMR**	r	0.136	0.095	0.029	0.117	0.149	0.102	−0.048	0.245	0.008	0.143	0.003	−0.068	0.133	0.062	0.309
	*p*	0.039	0.226	0.659	0.095	0.024	0.122	0.479	<0.001	0.908	0.031	0.964	0.304	0.059	0.413	<0.001
**PLR**	r	−0.052	−0.117	0.043	−0.019	−0.048	−0.131	0.110	−0.150	−0.019	−0.182	−0.051	0.061	−0.111	0.000	−0.265
	*p*	0.428	0.130	0.510	0.783	0.459	0.044	0.099	0.024	0.772	0.005	0.440	0.349	0.108	>0.999	<0.001
**AST/ALT ratio**	r	0.024	−0.130	0.036	0.034	0.145	−0.223	0.001	0.075	−0.019	−0.245	−0.016	0.205	−0.181	0.021	−0.042
	*p*	0.717	0.091	0.582	0.627	0.026	<0.001	0.991	0.257	0.770	<0.001	0.810	0.002	0.009	0.778	0.524
**CRP**	r	−0.243	0.002	−0.094	−0.220	−0.330	0.010	0.032	−0.398	−0.016	−0.113	0.094	−0.073	−0.074	−0.197	−0.397
	*p*	<0.001	0.983	0.152	0.001	<0.001	0.875	0.632	<0001	0.817	0.086	0.154	0.269	0.294	0.008	<0.001
**ESR**	r	−0.146	−0.012	0.009	−0.168	−0.262	−0.008	0.047	−0.316	0.011	−0.163	0.079	−0.083	−0.113	−0.075	−0.348
	*p*	0.035	0.881	0.896	0.021	<0.001	0.914	0.508	<0.001	0.872	0.018	0.253	0.231	0.126	0.339	<0.001
**ESR/CRP ratio**	r	0.104	−0.055	0.053	0.107	0.116	−0.028	−0.005	0.152	−0.012	0.021	−0.071	−0.017	0.004	0.083	0.161
	*p*	0.131	0.496	0.443	0.142	0.093	0.687	0.949	0.030	0.863	0.758	0.307	0.804	0.958	0.287	0.020
**CAR**	r	−0.262	0.015	−0.110	−0.215	−0.349	0.005	0.035	−0.409	−0.025	−0.122	0.116	−0.070	−0.074	−0.186	−0.406
	*p*	<0.001	0.851	0.099	0.002	<0.001	0.934	0.607	<0.001	0.710	0.067	0.082	0.295	0.295	0.014	<0.001
**Fibrinogen**	r	−0.156	−0.103	−0.006	−0.141	−0.198	−0.112	0.069	−0.257	0.007	−0.167	−0.030	−0.091	−0.175	−0.103	−0.217
	*p*	0.063	0.301	0.946	0.115	0.018	0.183	0.422	0.002	0.938	0.048	0.728	0.283	0.053	0.292	0.010
**D-dimer**	r	−0.113	0.009	0.026	−0.046	−0.222	−0.044	0.108	−0.307	0.054	−0.144	0.134	−0.015	−0.080	−0.101	−0.300
	*p*	0.102	0.910	0.703	0.531	0.001	0.527	0.121	<0.001	0.435	0.036	0.052	0.829	0.277	0.198	<0.001
**FAR**	r	−0.294	−0.079	−0.101	−0.154	−0.368	−0.110	0.078	−0.443	−0.041	−0.211	0.071	−0.098	−0.169	−0.133	−0.415
	*p*	<0.001	0.436	0.241	0.088	<0.001	0.199	0.369	<0.001	0.637	0.013	0.410	0.252	0.065	0.177	<0.001
**DFR**	r	−0.167	−0.028	−0.082	0.019	−0.132	−0.166	0.096	−0.210	−0.062	−0.151	0.136	0.107	−0.172	−0.037	−0.249
	*p*	0.052	0.782	0.346	0.837	0.126	0.055	0.273	0.015	0.481	0.081	0.118	0.220	0.065	0.713	0.004

Abbreviations: AST/ALT: aspartate aminotransferase/alanine aminotransferase; CAR: C-reactive protein/albumin ratio; CRP: C-reactive protein; DFR: D-dimer/fibrinogen ratio; ESR: erythrocyte sedimentation rate; FAR: fibrinogen/albumin ratio; HDL: high-density lipoprotein; IDL: intermediate-density lipoprotein; LDL: low-density lipoprotein; LMR: lymphocyte/monocyte ratio; MPV: mean platelet volume; NLR: neutrophil/lymphocyte ratio; PLR: platelet/lymphocyte ratio; VLDL: very-low-density lipoprotein; WBCs: white blood cells.

**Table 5:** Associations between outcome parameters and inflammatory parameters in simple logistic regression analysis.

	WBC			Monocytes			Lymphocytes		
	*OR*	*95% CI*	*p-value*	*OR*	*95% CI*	*p-value*	*OR*	*95% CI*	*p-value*
Any cardiovascular event	1.68	0.48–5.84	0.414	2.15	0.83–5.61	0.116	1.20	0.49–2.93	0.691
Death	1.40	0.19–10.18	0.740	0.75	0.17–3.35	0.702	0.26	0.06–1.12	0.070
Any glucocorticoid adverse effect	0.56	0.14–2.34	0.428	0.59	0.20–1.79	0.353	0.77	0.27–2.15	0.616
Newly developed relapse	**26.50**	**3.55–198.08**	**0.001**	1.51	0.39–5.90	0.552	1.04	0.30–3.61	0.948
	**Neutrophils**			**Thrombocytes**			**MPV**		
	*OR*	*95% CI*	*p-value*	*OR*	*95% CI*	*p-value*	*OR*	*95% CI*	*p-value*
Any cardiovascular event	1.12	0.45–2.80	0.803	0.94	0.23–3.76	0.927	6.09	0.12–302.52	0.364
Death	1.10	0.24–5.18	0.901	0.73	0.08–6.82	0.785	1.27	0.00–646.08	0.940
Any glucocorticoid adverse effect	1.10	0.38–3.19	0.860	0.58	0.12–2.88	0.503	0.36	0.00–31.32	0.651
Newly developed relapse	**7.21**	**1.73–30.15**	**0.007**	5.57	0.75–41.36	0.093	0.04	0.00–8.59	0.238
	**NLR**			**LMR**			**PLR**		
	*OR*	*95% CI*	*p-value*	*OR*	*95% CI*	*p-value*	*OR*	*95% CI*	*p-value*
Any cardiovascular event	0.93	0.50–1.74	0.825	0.73	0.34–1.56	0.413	0.84	0.37–1.92	0.672
Death	2.15	0.72–6.42	0.170	0.45	0.13–1.61	0.221	2.86	0.75–11.02	0.126
Any glucocorticoid adverse effect	1.26	0.61–2.60	0.538	1.07	0.44–2.58	0.882	1.03	0.40–2.68	0.947
Newly developed relapse	2.13	0.84–5.41	0.111	0.95	0.32–2.82	0.927	1.75	0.55–5.54	0.343
	**AST/ALT Ratio**			**CRP**			**ESR**		
	*OR*	*95% CI*	*p-value*	*OR*	*95% CI*	*p-value*	*OR*	*95% CI*	*p-value*
Any cardiovascular event	0.95	0.36–2.49	0.917	1.02	0.73–1.44	0.894	0.95	0.62–1.45	0.797
Death	0.76	0.16–3.62	0.733	1.01	0.58–1.74	0.974	0.98	0.51–1.89	0.944
Any glucocorticoid adverse effect	0.79	0.26–2.36	0.665	1.20	0.81–1.79	0.362	1.16	0.69–1.81	0.659
Newly developed relapse	0.44	0.11–1.75	0.241	0.79	0.49–1.29	0.350	1.11	0.61–2.03	0.732
	**ESR/CRP Ratio**			**CAR**			**Fibrinogen**		
	*OR*	*95% CI*	*p-value*	*OR*	*95% CI*	*p-value*	*OR*	*95% CI*	*p-value*
Any cardiovascular event	0.90	0.61–1.31	0.568	0.98	0.70–1.38	0.904	2.29	0.19–27.66	0.515
Death	0.95	0.53–1.71	0.872	1.05	0.61–1.81	0.853	4.97	0.22–113.46	0.315
Any glucocorticoid adverse effect	0.88	0.57–1.35	0.548	1.18	0.80–1.75	0.413	2.12	0.10–46.09	0.632
Newly developed relapse	1.25	0.74–2.11	0.398	0.81	0.50–1.30	0.373	1.37	0.06–30.72	0.842
	**D-Dimer**			**FAR**			**DFR**		
	*OR*	*95% CI*	*p-value*	*OR*	*95% CI*	*p-value*	*OR*	*95% CI*	*p-value*
Any cardiovascular event	1.03	0.62–1.72	0.917	0.53	0.04–6.77	0.628	1.06	0.55–2.03	0.863
Death	1.31	0.59–2.90	0.501	17.86	0.67–468.78	0.086	1.35	0.55–3.31	0.515
Any glucocorticoid adverse effect	0.77	0.43–1.39	0.388	0.98	0.05–19.62	0.990	0.57	0.26–1.27	0.169
Newly developed relapse	1.03	0.51–2.05	0.944	1.34	0.05–33.51	0.860	0.95	0.41–2.20	0.898

Abbreviations: AST/ALT: aspartate aminotransferase/alanine aminotransferase; CAR: C-reactive protein/albumin ratio; CRP: C-reactive protein; DFR: D-dimer/fibrinogen ratio; ESR: erythrocyte sedimentation rate; FAR: fibrinogen/albumin ratio; LMR: lymphocyte/monocyte ratio; MPV: mean platelet volume; NLR: neutrophil/lymphocyte ratio; PLR: platelet/lymphocyte ratio; WBCs: white blood cells.

## Data Availability

The data that support the findings of this study are available from the corresponding author upon request.

## Supplementary Materials

The supporting information can be downloaded at https://www.mdpi.com/article/10.3390/ijms26147016/s1.

## Abbreviations

The following abbreviations are used in this manuscript:

ACR	American College of Rheumatology
ADMA	Asymmetric dimethylarginine
Aix	Augmentation index
ALT	Alanine aminotransferase
AST	Aspartate aminotransferase
CAD	Coronary artery disease
CAR	CRP-to-albumin ratio
CRP	C-reactive protein
CT	Computed tomography
CVAD	Carotid and vertebral artery disease
DFR	D-dimer-to-fibrinogen ratio
ESC	European Society of Cardiology
ESR	Erythrocyte sedimentation rate
EULAR	European Alliance of Associations for Rheumatology
FAR	Fibrinogen-to-albumin ratio
GCA	Giant-cell arteritis
HDL	High-density lipoprotein
IDL	Intermediate-density lipoprotein
IMT	Intima-media thickness
LDL	Low-density lipoprotein
LEAD	Lower extremity artery disease
LMR	Lymphocyte-to-monocyte ratio
MAD	Mesenteric artery disease
MPV	Mean platelet volume
NLR	Neutrophil-to-lymphocyte ratio
OR	Odds ratio
PLR	Platelet-to-lymphocyte ratio
PMR	Polymyalgia rheumatic
PWV	Pulse-wave velocity
RAD	Renal artery disease
SDMA	Symmetric dimethylarginine
UEAD	Upper extremity arterial disease
VLDL	Very-low-density lipoprotein
VSMCs	Vascular smooth muscle cells
VTE	Venous thromboembolism
WBC	White blood count

## References

[1] Galassi F.M., Rühli F.J. (2016). A case of temporal arteritis in Filippino Lippi’s (1459–1504) Saint Frediano?. Clin. Rheumatol..

[2] Galassi F.M., Galassi S. (2016). A case of Horton’s disease (with its potential neurological symptoms) depicted in a portrait by Andrea Mantegna. Neurol. Sci..

[3] de Boysson H., Aouba A. (2022). An Updated Review of Cardiovascular Events in Giant Cell Arteritis. J. Clin. Med..

[4] Weyand C.M., Goronzy J.J. (2023). Immunology of Giant Cell Arteritis. Circ. Res..

[5] Weyand C.M., Liao Y.J., Goronzy J.J. (2012). The immunopathology of giant cell arteritis: Diagnostic and therapeutic implications. J. Neuroophthalmol..

[6] Rathore S.S., Srikaram P., Gudena S., Manoj S., Allam S.R., Hatamleh M.A., Naveen Chodisetti N.S., Shaikh S.P., Saravanan C.R., Woldehana N.A. (2025). Risk of cardiovascular events in giant cell arteritis: Systematic review and meta-analysis. Hellenic. J. Cardiol..

[7] Aouba A., Gonzalez Chiappe S., Eb M., Delmas C., de Boysson H., Bienvenu B., Rey G., Mahr A. (2018). Mortality causes and trends associated with giant cell arteritis: Analysis of the French national death certificate database (1980–2011). Rheumatology.

[8] Proven A., Gabriel S.E., Orces C., O’Fallon W.M., Hunder G.G. (2003). Glucocorticoid therapy in giant cell arteritis: Duration and adverse outcomes. Arthritis Rheum..

[9] Schweiger L., Hafner F., Meinitzer A., Brodmann M., Dejaco C., Jud P. (2024). Association of clinical, imaging and laboratory parameters with adverse effects of glucocorticoid therapy in patients with giant cell arteritis. Front. Med..

[10] Dart A.M., Chin-Dusting J.P. (1999). Lipids and the endothelium. Cardiovasc. Res..

[11] Lo Gullo A., Giuffrida C., Morace C., Squadrito G., Magnano San Lio P., Ricciardi L., Salvarani C., Mandraffino G. (2022). Arterial Stiffness and Adult Onset Vasculitis: A Systematic Review. Front. Med..

[12] Schlesinger S., Sonntag S.R., Lieb W., Maas R. (2016). Asymmetric and Symmetric Dimethylarginine as Risk Markers for Total Mortality and Cardiovascular Outcomes: A Systematic Review and Meta-Analysis of Prospective Studies. PLoS ONE.

[13] Hellmich B., Agueda A., Monti S., Buttgereit F., de Boysson H., Brouwer E., Cassie R., Cid M.C., Dasgupta B., Dejaco C. (2020). 2018 Update of the EULAR recommendations for the management of large vessel vasculitis. Ann. Rheum. Dis..

[14] Maz M., Chung S.A., Abril A., Langford C.A., Gorelik M., Guyatt G., Archer A.M., Conn D.L., Full K.A., Grayson P.C. (2021). 2021 American College of Rheumatology/Vasculitis Foundation Guideline for the Management of Giant Cell Arteritis and Takayasu Arteritis. Arthritis Rheumatol..

[15] Kermani T.A., Warrington K.J., Cuthbertson D., Carette S., Hoffman G.S., Khalidi N.A., Koening C.L., Langford C.A., Maksimowicz-McKinnon K., McAlear C.A. (2015). Disease Relapses among Patients with Giant Cell Arteritis: A Prospective, Longitudinal Cohort Study. J. Rheumatol..

[16] Aydın C., Uyan U., Karadeniz M., Demirkıran A. (2023). Role of simple inflammatory parameters in predicting the severity of coronary artery disease. Rev. Assoc. Med. Bras..

[17] Oh L.J., Wong E., Andrici J., McCluskey P., Smith J.E.H., Gill A.J. (2018). Full blood count as an ancillary test to support the diagnosis of giant cell arteritis. Intern. Med. J..

[18] Pan L., Du J., Li T., Liao H. (2017). Platelet-to-lymphocyte ratio and neutrophil-to-lymphocyte ratio associated with disease activity in patients with Takayasu’s arteritis: A case-control study. BMJ Open.

[19] Seringec Akkececi N., Yildirim Cetin G., Gogebakan H., Acipayam C. (2019). The C-Reactive Protein/Albumin Ratio and Complete Blood Count Parameters as Indicators of Disease Activity in Patients with Takayasu Arteritis. Med. Sci. Monit..

[20] Gasparyan A.Y., Ayvazyan L., Mukanova U., Yessirkepov M., Kitas G.D. (2019). The Platelet-to-Lymphocyte Ratio as an Inflammatory Marker in Rheumatic Diseases. Ann. Lab. Med..

[21] Wu Y., Chen Y., Yang X., Chen L., Yang Y. (2016). Neutrophil-to-lymphocyte ratio (NLR) and platelet-to-lymphocyte ratio (PLR) were associated with disease activity in patients with systemic lupus erythematosus. Int. Immunopharmacol..

[22] Galli E., Muratore F., Mancuso P., Boiardi L., Marvisi C., Besutti G., Spaggiari L., Casali M., Versari A., Giorgi Rossi P. (2022). The role of PET/CT in disease activity assessment in patients with large vessel vasculitis. Rheumatology.

[23] Littlejohn E., Marder W., Lewis E., Francis S., Jackish J., McCune W.J., Somers E.C. (2018). The ratio of erythrocyte sedimentation rate to C-reactive protein is useful in distinguishing infection from flare in systemic lupus erythematosus patients presenting with fever. Lupus.

[24] Zhou W., Li X., Jiang Y., Li S., Jin M., Sui J., Wang J. (2021). The Significance of Blood Index and Biochemistry Index in Patients with Rheumatoid Arthritis. Clin. Lab..

[25] Sundermann A.C., Saum K., Conrad K.A., Russell H.M., Edwards T.L., Mani K., Björck M., Wanhainen A., Owens A.P. (2018). Prognostic value of D-dimer and markers of coagulation for stratification of abdominal aortic aneurysm growth. Blood Adv..

[26] Zhang D.P., Mao X.F., Wu T.T., Chen Y., Hou X.G., Yang Y., Ma X., Zhang J.Y., Ma Y.T., Xie X. (2020). The Fibrinogen-to-Albumin Ratio Is Associated with Outcomes in Patients with Coronary Artery Disease Who Underwent Percutaneous Coronary Intervention. Clin. Appl. Thromb. Hemost..

[27] Nadkarni S., Lashin H., Hollywood J., Dasgupta B., Mason J.C., Perretti M. (2019). Identification of an activated neutrophil phenotype in polymyalgia rheumatica during steroid treatment: A potential involvement of immune cell cross-talk. Clin. Sci..

[28] Jung J.Y., Lee E., Suh C.H., Kim H.A. (2019). Neutrophil-to-lymphocyte ratio and platelet-to-lymphocyte ratio are associated with disease activity in polymyalgia rheumatica. J. Clin. Lab. Anal..

[29] Chen H., Tan C., Wang Z., Zha J., Liu H., Dong Z., Chen G. (2023). Long-term glucocorticoid exposure persistently impairs CD4+ T cell biology by epigenetically modulating the mTORC1 pathway. Biochem. Pharmacol..

[30] Quatrini L., Tumino N., Besi F., Ciancaglini C., Galaverna F., Grasso A.G., Merli P., Locatelli F., Vacca P., Moretta L. (2022). Glucocorticoids inhibit human hematopoietic stem cell differentiation toward a common ILC precursor. J. Allergy Clin. Immunol..

[31] Karcıoğlu Batur L., Savaş S., Girgin E., Hekim N. (2022). Association of the IL-6R gene polymorphic variant rs2228145(C>A) with IL-6 gene polymorphisms in a healthy cohort of Turkish population. Genes Immun..

[32] Rizzo C., La Barbera L., Miceli G., Tuttolomondo A., Guggino G. (2022). The innate face of Giant Cell Arteritis: Insight into cellular and molecular innate immunity pathways to unravel new possible biomarkers of disease. Front. Mol. Med..

[33] Vanhoutte P.M., Zhao Y., Xu A., Leung S.W. (2016). Thirty Years of Saying NO: Sources, Fate, Actions, and Misfortunes of the Endothelium-Derived Vasodilator Mediator. Circ. Res..

[34] Bhat T., Teli S., Rijal J., Bhat H., Raza M., Khoueiry G., Meghani M., Akhtar M., Costantino T. (2013). Neutrophil to lymphocyte ratio and cardiovascular diseases: A review. Expert. Rev. Cardiovasc. Ther..

[35] Yüksel M., Yıldız A., Oylumlu M., Akyüz A., Aydın M., Kaya H., Acet H., Polat N., Bilik M.Z., Alan S. (2015). The association between platelet/lymphocyte ratio and coronary artery disease severity. Anatol. J. Cardiol..

[36] Dimitroulas T., Hodson J., Sandoo A., Smith J., Kitas G.D. (2017). Endothelial injury in rheumatoid arthritis: A crosstalk between dimethylarginines and systemic inflammation. Arthritis Res. Ther..

[37] Ding Y., Xue L. (2022). The potential value of fibrinogen to albumin ratio (FAR) in the assessment of inflammation in spondyloarthritis. BMC Musculoskelet. Disord..

[38] Iqbal T., Raza N., Marwat Z.I., Riyaz A. (2011). Inflammatory markers and lipid profile in patients of coronary artery disease. J. Ayub Med. Coll. Abbottabad..

[39] Giannakopoulou S.P., Antonopoulos A., Panagiotakos D. (2024). Serum Inflammatory Markers Used in Cardiovascular Disease Risk Prediction Models: A Systematic Review. Angiology.

[40] Li Y., Zhong X., Cheng G., Zhao C., Zhang L., Hong Y., Wan Q., He R., Wang Z. (2017). Hs-CRP and all-cause, cardiovascular, and cancer mortality risk: A meta-analysis. Atherosclerosis.

[41] Liu Y., Guan S., Xu H., Zhang N., Huang M., Liu Z. (2023). Inflammation biomarkers are associated with the incidence of cardiovascular disease: A meta-analysis. Front. Cardiovasc. Med..

[42] Jud P., Verheyen N., Dejaco C., Haas E., Szolar D., Meinitzer A., Duftner C., Thonhofer R., Gressenberger P., Brodmann M. (2021). Prevalence and prognostic factors for aortic dilatation in giant cell arteritis—A longitudinal study. Semin. Arthritis Rheum..

[43] Dejaco C., Duftner C., Buttgereit F., Matteson E.L., Dasgupta B. (2017). The spectrum of giant cell arteritis and polymyalgia rheumatica: Revisiting the concept of the disease. Rheumatology.

[44] Aboyans V., Ricco J.B., Bartelink M.E.L., Björck M., Brodmann M., Cohnert T., Collet J.P., Czerny M., De Carlo M., Debus S. (2018). ESC Scientific Document Group. 2017 ESC Guidelines on the Diagnosis and Treatment of Peripheral Arterial Diseases, in collaboration with the European Society for Vascular Surgery (ESVS): Document covering atherosclerotic disease of extracranial carotid and vertebral, mesenteric, renal, upper and lower extremity arteries. Endorsed by: The European Stroke Organization (ESO)The Task Force for the Diagnosis and Treatment of Peripheral Arterial Diseases of the European Society of Cardiology (ESC) and of the European Society for Vascular Surgery (ESVS). Eur. Heart J..

[45] Williams B., Mancia G., Spiering W., Agabiti Rosei E., Azizi M., Burnier M., Clement D.L., Coca A., de Simone G., Dominiczak A. (2018). ESC Scientific Document Group 2018 ESC/ESH Guidelines for the management of arterial hypertension. Eur. Heart J..

[46] Rogers I.S., Massaro J.M., Truong Q.A., Mahabadi A.A., Kriegel M.F., Fox C.S., Thanassoulis G., Isselbacher E.M., Hoffmann U., O’Donnell C.J. (2013). Distribution, determinants, and normal reference values of thoracic and abdominal aortic diameters by computed tomography (from the Framingham Heart Study). Am. J. Cardiol..

[47] Jud P., Hafner F., Meinitzer A., Brodmann M., Dejaco C., Silbernagel G. (2023). Cardiovascular diseases and their associations with lipid parameters and endothelial dysfunction in giant cell arteritis. RMD Open.

