# Supplementary material for: Correlation and Risk Assessment of Inflammation-Based Parameters on Cardiovascular Parameters and Clinical Events in Giant Cell Arteritis: A Retrospective Study
Source: Int J Mol Sci. 2025 Jul 21;26(14):7016. doi: 10.3390/ijms26147016 (PMC12295461; doi:10.3390/ijms26147016)
Supplement: Supplementary file 1 [file ijms-26-07016-s001.zip › ijms-3679569-supplementary.pdf]

Supp. Table 1: Patients' characteristics at study inclusion and retrospectively collected cardiovascular parameters and potential glucocorticoid adverse effects

	**GCA (n=138)**
Age (years), mean (± SD)	74.5 (±7.7)
Sex, n (%)	
Female	106 (76.8)
Male	32 (23.2)
BMI (kg/m²), mean (± SD)	26.47 (±4.65)
GCA subtype, n (%)	
GCA without PMR	77 (55.8)
Cranial GCA	69 (50.0)
Extracranial GCA	8 (5.8)
GCA with PMR	61 (44.2)
Prevalent cardiovascular risk factors, n (%)	
Arterial hypertension	97 (70.3)
Diabetes mellitus	28 (20.3)
Obesity	24 (17.4)
Hyperlipidemia	92 (66.7)
Hypercholesterolemia	85 (61.6)
Hypertriglyceridemia	41 (29.7)
Smoking	31 (23.9)
Active smoking	17 (12.3)
Ex-smoker	16 (11.6)
Prevalent cardiovascular disease, n (%)	
CAD	24 (17.4)
Asymptomatic	14 (10.1)
MCI	10 (7.2)
CVAD	81 (58.7)
Asymptomatic	66 (47.8)
Stroke/TIA	15 (10.9)
UEAD	14 (10.1)
Asymptomatic	8 (5.8)
Intermittent claudication	5 (3.6)
Upper limb CLTI	1 (0.7)
Acute upper limb ischemia	2 (1.4)
LEAD	25 (18.1)
Asymptomatic	13 (9.4)
Intermittent claudication	11 (8.0)
Lower limb CLTI	1 (0.7)
Acute lower limb ischemia	1 (0.7)
RAD	2 (1.4)
Asymptomatic	1 (0.7)
Symptomatic	1 (0.7)
MAD	3 (2.2)

Asymptomatic	3 (2.2)
Symptomatic	0 (0.0)
Aortic damage, n (%)	
Aortic dilatation/aneurysm	
Ascending aorta	27 (19.6)
Thoracic descending aorta	25 (18.1)
Infrarenal abdominal aorta	9 (6.5)
Aortic dissection	0 (0.0)
Concomitant drug therapy, n (%)	
Antiplatelet therapy	73 (52.9)
Oral anticoagulation	17 (12.3)
ACE inhibitors	43 (31.2)
Beta blockers	57 (41.3)
Calcium channel blockers	12 (8.7)
Diuretics	24 (17.4)
Other antihypertensives	15 (10.9)
Insulin	4 (2.9)
Metformin	12 (8.7)
Statins	45 (32.6)
DMARD	18 (13.0)
Methotrexate	15 (10.9)
Azathioprine	3 (2.2)
Prevalent potential glucocorticoid adverse effects, n (%)	
CKD	129 (93.5)
CKD 1	0 (0.0)
CKD 2	69 (49.3)
CKD 3	57 (41.3)
CKD 3a	48 (34.9)
CKD 3b	9 (6.5)
CKD 4	4 (2.9)
CKD 5	0 (0.0)
Osteoporosis	71 (51.4)
Bone fracture	25 (18.1)
Cataract	44 (31.9)
Glaucoma	13 (9.4)
Hepatic steatosis	14 (10.1)
Hepatic cirrhosis	0 (0.0)
VTE	12 (8.7)
Depression	8 (5.8)
Dementia	4 (2.9)
Gastritis	28 (20.3)
Peptic ulcer	6 (4.3)
Esophagitis	18 (13.0)
Pancreatitis	6 (4.3)
Aortic size (mm), mean (± SD)	

Ascending aorta	34.7 (±4.6)
Thoracic descending aorta	25.8 (±3.3)
Infrarenal abdominal aorta	17.3 (±2.4)

Abbreviations: ACE: angiotensin-converting enzyme; BMI: body mass index; CAD: coronary artery disease; CLTI: chronic limb threatening ischemia; CKD: chronic kidney disease; CVAD: carotid and vertebral artery disease; DMARD: disease-modified antirheumatic drug; GCA: giant cell arteritis; LEAD: lower extremity artery disease; MAD: mesenteric artery disease; MCI: myocardial infarction; PMR: polymyalgia rheumatica; RAD: renal artery disease; TIA: transient ischemic attack; UEAD: upper extremity artery disease; VTE: venous thromboembolism

Supp. Table 2: Development of cardiovascular outcome parameters, glucocorticoid adverse effects and relapse during the follow-up period.

	**GCA (n=138)**
Relapse, n (%)	22 (15.9)
Minor relapse	17 (12.3)
Major relapse	5 (3.6)
Any new cardiovascular event, n (%)	70 (50.7)
New cardiovascular events per patient (n), median (25th-75th percentile)	1 (0-1)
CAD, n (%)	25 (18.1)
Asymptomatic	12 (8.7)
MCI	13 (9.4)
CVAD, n (%)	31 (22.5)
Asymptomatic	14 (10.1)
Stroke/TIA	17 (12.3)
UEAD, n (%)	9 (6.5)
Asymptomatic	9 (6.5)
Intermittent claudication	0 (0.0)
Upper limb CLTI	0 (0.0)
Acute upper limb ischemia	0 (0.0)
Any Fontaine stage progression of previous UEAD	0 (0.0)
LEAD, n (%)	17 (12.3)
Asymptomatic	14 (10.1)
Intermittent claudication	1 (0.7)
Lower limb CLTI	3 (2.2)
Acute lower limb ischemia	6 (4.3)
Any Fontaine stage progression of previous LEAD	8 (5.8)
RAD, n (%)	16 (11.6)
Asymptomatic	15 (10.9)
Symptomatic	1 (0.7)
MAD, n (%)	12 (8.7)
Asymptomatic	12 (8.7)
Symptomatic	0 (0.0)
Aortic rupture, n (%)	0 (0.0)
Aortic dissection, n (%)	0 (0.0)
Aortic repair, n (%)	1 (0.7)
Death, n (%)	15 (10.9)
Cardiovascular death	3 (2.2)
Patients with any potentially new glucocorticoid adverse effect, n (%)	104 (75.4)
Number of potentially new glucocorticoid adverse effects, median (25th-75th percentile)	1 (0-3)
Potential new glucocorticoid adverse effects, n (%)	
Arterial hypertension	17 (12.3)
Diabetes mellitus	5 (3.6)

Obesity	4 (2.9)
Hyperlipidemia	15 (10.9)
Hypercholesterolemia	13 (9.4)
Hypertriglyceridemia	10 (7.2)
New CKD	6 (4.3)
CKD stage progression	40 (29.0)
CKD 1	3 (2.2)
CKD 2	69 (50.0)
CKD 3	56 (40.6)
CKD 3a	38 (27.5)
CKD 3b	18 (13.0)
CKD 4	6 (4.3)
CKD 5	1 (0.7)
Osteoporosis	10 (7.2)
Bone fracture	32 (23.2)
Cataract	25 (18.1)
Glaucoma	3 (2.2)
Hepatic steatosis	3 (2.2)
Hepatic cirrhosis	0 (0.0)
VTE	10 (7.2)
Depression	5 (3.6)
Dementia	17 (12.3)
Gastritis	7 (5.1)
Peptic ulcer	1 (0.7)
Esophagitis	6 (4.3)
Pancreatitis	5 (3.6)

Abbreviations: CAD: coronary artery disease; CKD: chronic kidney disease; CLTI: chronic limb threatening ischemia; CVAD: carotid and vertebral artery disease; GCA: giant cell arteritis; LEAD: lower extremity artery disease; MAD: mesenteric artery disease; MCI: myocardial infarction; RAD: renal artery disease; TIA: transient ischemic attack; UEAD: upper extremity artery disease; VTE: venous thromboembolism
